# Selective antitumor activity of roscovitine in head and neck cancer

**DOI:** 10.18632/oncotarget.9560

**Published:** 2016-05-23

**Authors:** Cyril Gary, Michael Hajek, Asel Biktasova, Gary Bellinger, Wendell G. Yarbrough, Natalia Issaeva

**Affiliations:** ^1^ Department of Surgery Division of Otolaryngology, Yale University, New Haven, CT USA; ^2^ Department of Pathology, Yale University, New Haven, CT USA; ^3^ Department of Yale Cancer Center, Yale University, New Haven, CT USA; ^4^ Current address: Children's Cancer Institute, Lowy Cancer Research Centre, UNSW, Australia

**Keywords:** roscovitine, head and neck cancer, HPV, DNA damage, toxicity

## Abstract

Radiation and chemotherapy that are commonly used to treat human cancers damage cellular DNA. DNA damage appears to be more toxic to cancer cells than normal cells, most likely due to deregulated checkpoint activation and/or deficiency in DNA repair pathways that are characteristics of many tumors. However, unwanted side effects arise as a result of DNA damage to normal cells during the treatment.

Here, we show that roscovitine, a cyclin-dependent kinase (CDK) inhibitor that inhibits CDK-1, CDK-2, CDK-5, CDK-7, and CDK-9 due to competitive binding to the ATP site on the kinases, causes significant DNA damage followed by p53-dependent cell death in human papilloma virus (HPV)-positive, but not in HPV-negative, head and neck cancer cells. Since HPV positivity was a molecular marker for increased sensitivity of cells to roscovitine, we reasoned that systemic roscovitine administration would not be toxic to healthy HPV-negative tissue. Indeed, low roscovitine doses significantly inhibited the growth of HPV-associated xenografted tumors in mice without causing any detectable side effects.

Given that inhibition of CDKs has been shown to inhibit replication of several viruses, we suggest that roscovitine treatment may represent a selective and safe targeted therapeutic option against HPV-positive head and neck cancer.

## INTRODUCTION

Head and neck squamous cell carcinoma (HNSCC) is the eighth most prevalent type of cancer in the world. Although incidence rates of HNSCC have been steadily declining from the 1980s to present, there has been an ominous rise in the incidence of a particular subset of HNSCC during the same time period: oropharyngeal squamous cell carcinoma (OPSCC) [1, 2]. About 70% of OPSCCs are associated with HPV, an 8kb double stranded DNA virus that has been classically known as the primary etiological agent of cervical cancer, and is now considered a major cause of OPSCC [3, 4]. Although the prevalence of HPV in HNSCC is relatively lower overall (estimated to be around 20%) than that found in OPSCC, HPV status nonetheless is now considered a major risk factor for developing HNSCC along with the traditional risk factors of alcohol and tobacco use [5, 6]. The majority of HPV-positive (HPV+) OPSCCs are associated with the high-risk HPV16 strain, which is also the most common strain found in HPV+ cervical cancers [7, 8]. Patients with HPV+ OPSCC can be viewed as a separate population from HPV- HNSCC patients, partially because they have higher response to treatment, increased overall survival, lower risk of disease progression, and lower risk of recurrence in response to chemotherapy and radiation treatment [7–9]. However, even though HPV+ patients respond better to conventional therapies, they suffer from the deleterious side effects of chemotherapy and radiation, and are still at risk for developing chemotherapy resistance. According to the most recent National Comprehensive Cancer Network (NCCN) guidelines on head and neck cancer, the HPV status of a cancer should not change management decisions and treatment paradigms outside of clinical trials; rather, it is used for prognosis [10]. However, given the recent rise of HPV+ OPSCC and the apparent differences in underlying disease mechanisms between HPV-positive and HPV-negative OPSCCs, we sought to investigate whether a novel targeted therapy, aimed at exploiting the HPV status of HNSCC, could provide an effective treatment with less harmful side effects to patients.

Activation of CDKs appears to be the most important regulatory step in cell cycle progression. As their name implies, CDKs form complexes with cyclins and initiate a cascade of downstream signaling events that prompt the cell to synthesize DNA, initiate mitosis, and finally complete the cell cycle. Dysregulation of this process is frequently implicated in cancer. As such, small molecule CDK-inhibitors have become promising as potential targeted anti-neoplastic agents, with over a hundred different CDK-inhibitors currently in varying stages of clinical trials [11]. Roscovitine is one such CDK-inhibitor. It competes for the ATP-binding site of CDKs and primarily inhibits CDK2 along with CDK1, CDK5, CDK7, and CDK9 [12–14]. Roscovitine has been shown to have cytotoxic effects in numerous human cancer cell lines and is currently in phase II clinical trials for non-small-cell lung and nasopharyngeal cancers [15–17]. Besides the aforementioned antitumor properties, roscovitine was of particular interest to our study because: 1) roscovitine was shown to be highly cytotoxic towards HPV18 and HPV16-positive human cervical cancer cells [18, 19], and 2) roscovitine was shown to inhibit the replication and DNA synthesis of herpes simplex virus [18, 20]. In our study, roscovitine was found to be particularly cytotoxic to HPV+ HNSCC cell lines compared to HPV- head and neck cancer cells, suggesting that the sensitivity of HNSCC cells to roscovitine is dependent on HPV status and reinforcing the potential of roscovitine as a novel anti-HPV+ HNSCC agent. HPV+ HNSCC cells were particularly sensitive to roscovitine, because roscovitine treatment selectively induced DNA damage, thus triggering p53-dependent cell death in HPV+, but not in HPV- HNSCC cells. Finally, low doses of roscovitine significantly retarded the rate of tumor growth of HPV+ HNSCC cells *in vivo* without causing any apparent side effects. These findings all support the potential of roscovitine as a novel anti-HPV+ HNSCC agent.

## RESULTS

### Sensitivity of head and neck cancer cells to roscovitine depends on HPV status

**Figure 1:**
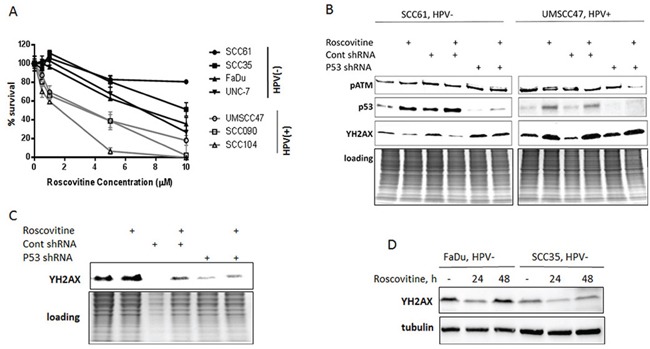
Roscovitine induces p53- and ATM-independent phosphorylation of H2AX and selectively inhibits growth in HPV-positive head and neck cancer cells **A.** Survival after increasing doses of roscovitine was determined in HPV-negative SCC35, SCC61, FaDu and UNC-7 (labeled in black) and HPV+ UMSCC47, SCC104 and SCC090 (labeled in grey) head and neck cancer cell lines; standard deviations are calculated from four independent experiments. **B.** HPV- SCC61 and HPV+ UMSCC47 cells expressing either control, or p53 shRNAs, were treated with 20μM of roscovitine; immunoblotting with indicated antibodies was performed 24 hours after the treatment. **C.** HPV+ SCC090 cells transfected with control or p53 shRNA were treated with roscovitine for 24h and immunoblotted with ϒH2AX antibody. **D.** Two HPV- cells lines, FaDu and SCC35, were treated with 20μM of roscovitine for 24 and 48 hours and immunoblotted with indicated antibodies.

Since previous studies suggested that cervical cancer cells were sensitive to roscovitine and experienced both significant inhibition of proliferation and increased caspase-mediated apoptosis in response to roscovitine treatment [18, 19], we first tested whether HPV status had an effect on the sensitivity of head and neck cancer cells to roscovitine. A survival assay was performed to gauge the response of four HPV-negative (SCC61, SCC35, FaDU, and UNC-7) and three HPV-positive (UMSCC47, SCC090 and SCC104) HNSCC cell lines to different roscovitine concentrations. As demonstrated in Figure 1A, the HPV+ cancer cell lines experienced significantly decreased clonogenic survival in response to roscovitine treatment in a dose-dependent manner, when compared to the HPV- cancer cell lines. Among HPV- cells, the sensitivity to roscovitine was not dependent on p53 mutation status, as there was no significant variations between wild type p53 expressing UNC7 cells and mutant p53 carrying SCC61, SCC35, and FaDU cell lines. Furthermore, the greatest differences between HPV+ and HPV- cancer cell lines roscovitine sensitivity were found at lower concentrations of roscovitine administered, reinforcing the therapeutic potential of roscovitine as a selective agent against HPV+ head and neck cancer cells.

### Roscovitine promotes p53- and ATM-independent stimulation of DNA damage response selectively in HPV+ head and neck cancer cells

**Figure 2:**
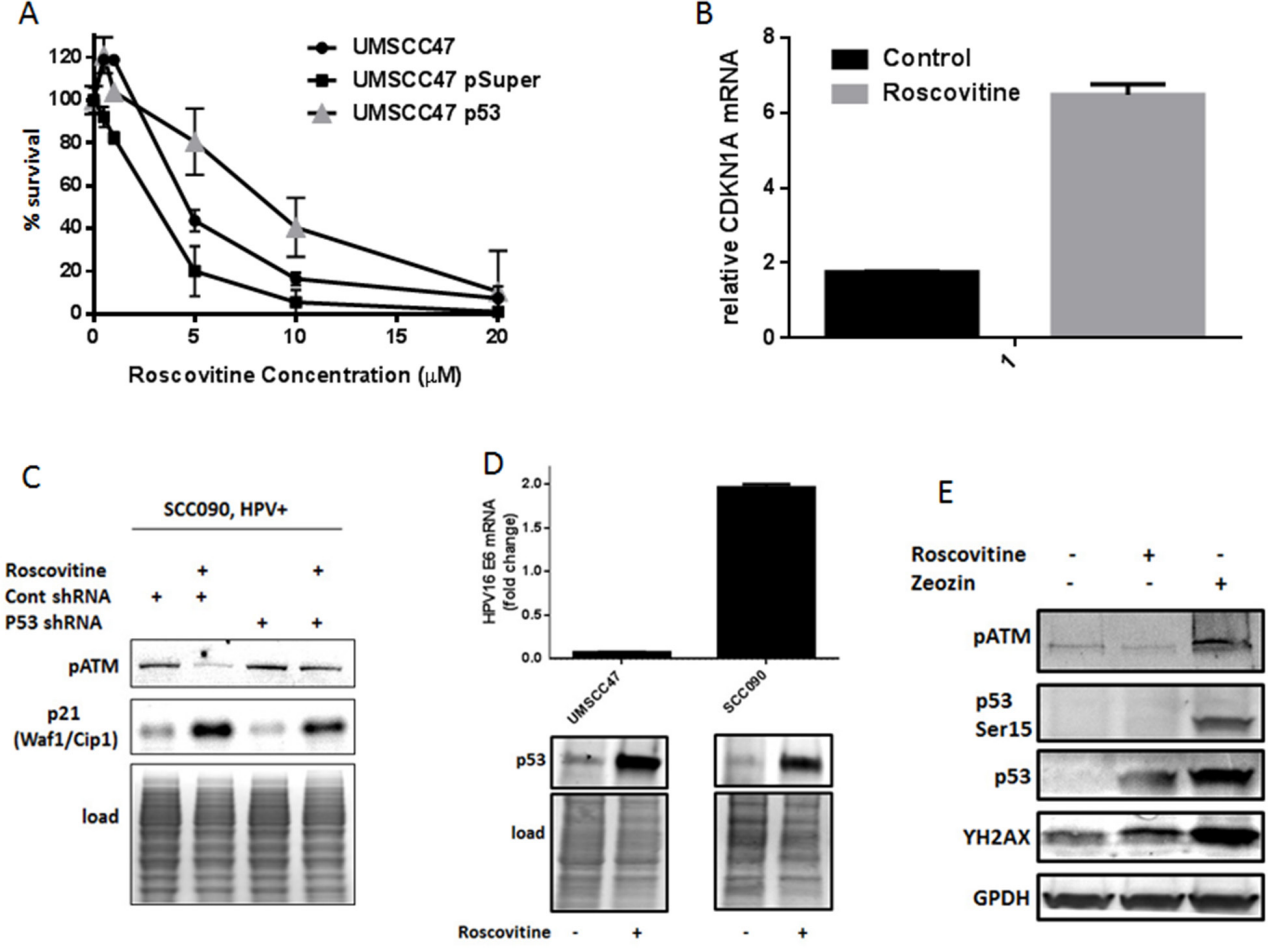
Roscovitine treatment activates p53 and induces p53-dependent suppression of HPV-positive cells growth **A.** HPV+ UMSCC47 cells were transiently transfected with control or p53 shRNA and plated for survival after the treatment with increasing doses of roscovitine; standard deviations are calculated from two independent experiments. **B.** Relative mRNA levels of p53 target gene CDKN1A (p21) in HPV-positive SCC090 cells treated or not with 20 μM roscovitine for 24 hours; standard deviations are calculated from two independent experiments. **C.** SCC090 cells were treated or not with roscovitine, lysed, and immunoblotted with indicated antibodies. **D.** HPV-positive UMSCC47 and SCC090 cells were treated or not with roscovitine; the cells were collected and HPV16 mRNA levels were determined in qRT-PCR (**top**), or p53 protein levels were determined in Western Blot (**bottom**). **E.** HPV-positive SCC090 cells were treated for 24 hours with roscovitine, or zeocin, or left untreated as a control, lysed and immunoblotted with indicated antibodies.

Given that HPV status confers sensitivity to roscovitine in head and neck cancer cells, we next investigated the potential mechanism of this sensitivity. Roscovitine has been shown to stabilize and activate wild-type p53 and induce apoptosis in multiple human cancer cell lines [21–23], including HPV18-positive cervical cancer HeLa cells [19]. Furthermore, roscovitine has been reported to activate DNA damage response pathways [24] and inhibit DNA damage repair machinery [25], although whether roscovitine treatment damages cellular DNA remains unclear. We found that roscovitine upregulates p53 in head and neck cancer cells regardless of p53 mutation and HPV status (Figure 1B; HPV-negative SCC61 cells harbor mutant p53, while HPV-positive UMSCC47 cells have wild type p53). Intriguingly, roscovitine activated DNA damage response, as detected by phosphorylation of H2AX (ϒH2AX), in HPV-positive UMSCC47 cells only (Figure 1B). In contrast, we found a significant decrease in H2AX phosphorylation in HPV-negative SCC61 head and neck cancer cells after roscovitine treatment (Figure 1B). Depletion of p53 with p53 shRNA neither abrogated ϒH2AX induction in HPV-positive, nor redaction of H2AX phosphorylation in HPV-negative cells (Figure 1B). Interestingly, DNA damage-responsive kinase, ATM, was not activated by roscovitine treatment in any cells tested (Figures 1B, 2C), suggesting that stimulation of DNA damage response by roscovitine treatment proceeded via an ATM-independent pathway in HPV-positive cells. Similar results were obtained in another HPV+ cell line, SCC090, in which roscovitine treatment resulted in activation of DNA damage response, as indicated by elevated phosphorylation of H2AX, independently of the presence or absence of p53 (Figure 1C). In addition, analogous to the results obtained with HPV- SCC61 cells, 24 hour treatment with roscovitine downregulated phosphorylation of H2AX in two other HPV-negative head and neck cancer cells, SCC35 and Fadu, with γH2AX levels restored back to control untreated cells 48 hours after the treatment (Figure 1D). Thus, roscovitine activated DNA damage response selectively in HPV+, but not in HPV- head and neck cancer cells.

### Roscovitine treatment activates p53 and induces p53-dependent HPV-positive cell death

The tumor suppressor wild type p53 is a powerful inducer of cell death in response to diverse stress signals, including DNA damage. In HPV-positive cancer cells, the HPV oncoprotein E6 induces degradation of p53 through ubiquitin-mediated proteolysis, leading to the loss of p53 activity. However, we found that roscovitine treatment elevated p53 levels in HPV+ head and neck cancer cells (Figure 1B). In order to determine whether the increased sensitivity of HPV+ cells to roscovitine is due to upregulation and activation of wild type p53, we transiently transfected UMSCC47 cells with psuper control or psuper p53 shRNAs. Indeed, depletion of p53 resulted in increased survival of UMSCC47 cells (Figure 2A) after roscovitine treatment. The classical p53 target gene, CDKN1A, was upregulated by roscovitine in HPV+ SCC090 cells on mRNA (Figure 2B) and protein levels (Figure 2C), suggesting that roscovitine-elevated p53 is transcriptionally active. Depletion of p53 with shRNA partially abolished p21 induction after roscovitine treatment (Figure 2C), further confirming roscovitine-induced p53 transcriptional activation.

Next, we attempted to find a mechanism of p53 induction in HPV-positive head and neck cancer cells after roscovitine treatment. First, we determined the expression of p53 negative regulator HPV E6. Interestingly, roscovitine treatment differently affected HPV E6 levels in two HPV-positive cell lines: while roscovitine decreased HPV E6 expression in UMSCC47 cells, it upregulated HPV E6 mRNA in SCC090 cell line (Figure 2D, top). Despite the opposite effect on HPV16 E6 mRNA levels, roscovitine treatment induced p53 protein in both cell lines (Figure 2D, bottom). Thus, p53 was upregulated by roscovitine independently of HPV E6 expression. To prove that roscovitine-induced DNA damage stabilized p53 in HPV-positive head and neck cancer cells, we treated SCC090 cells the with radiomimetic drug zeocin. As expected, zeocin induced DNA damage, as indicated by increased phosphorylation of H2AX, and upregulated p53 protein (Figure 2E). In contrast to roscovitine, zeocin activated ATM, resulting in phosphorylation of p53 at Ser15. Similar to UMSCC47 cells (Figure 1B), roscovitine triggered DNA damage response and upregulated the total level of p53, while it did not activate ATM and did not induce p53 phosphorylation at Ser15 in SCC090 cells (Figure 2E).

Together, our data suggested that roscovitine activates ATM-independent DNA damage response that stabilizes p53 and promotes p53-dependent cell death in HPV+ head and neck cancer cells.

### Roscovitine does not induce DNA double strand breaks as indicated by the absence of 53BP1 foci formation

**Figure 3:**
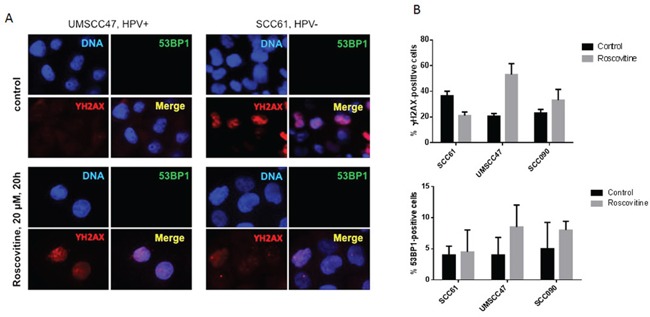
Roscovitine does not induce DNA double strand breaks as indicated by the absence of 53BP1 foci formation **A.** HPV- SCC61 and HPV+ UMSCC47 cells were treated with 20μM of roscovitine for 24 hours. Cells were fixed and immunostained with γH2AX and 53BP1 antibodies; representative images are shown. **B.** Quantification of γH2AX and 53BP1 positive cells from two independent experiments.

Phosphorylation of H2AX at Ser139 is commonly used as a marker for general DNA damage; it is also elevated in the process of apoptosis, during progression of replication forks, and in G2/M arrest [26–28]. To determine which particular events caused the phosphorylation of H2AX in HPV-positive head and neck cancer cells after roscovitine treatment, we tracked the formation of 53BP1 foci as a marker of DNA double strand breaks (DSBs) [29, 30]. Confirming our immunoblotting data, showing changes in H2AX phosphorylation (Figure 1B, 1C and 1D), treatment with roscovitine induced formation of γH2AX foci in HPV+ cells, UMSCC47, and SCC090, while reduced the number of γH2AX-positive cells in HPV- cell line SCC61 (Figure 3A and 3B). However, no significant differences in the number of 53BP1-positive cells in control untreated and roscovitine treated samples were found in any of cell lines tested (Figure 3A and 3B). This suggested that roscovitine treatment does not induce formation of DNA DSBs.

### Roscovitine induces RPA foci formation in HPV+, but not in HPV- head and neck cancer cells

**Figure 4:**
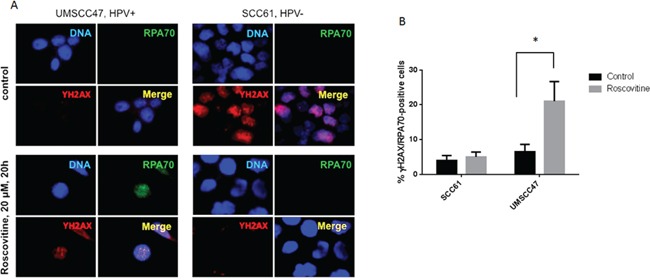
Roscovitine induces RPA foci formation in HPV+, but not in HPV-, head and neck cancer cells **A.** HPV- SCC61 and HPV+ UMSCC47 cells were treated with 20μM of roscovitine for 24 hours. Cells were fixed and immunostained with γH2AX and RPA70 antibodies; representative images are shown. **B.** Quantification of RPA-positive cells from two independent experiments.

Due to its strong affinity to single stranded DNA (SSD) and ability to attract other proteins to these sites, Replication Protein A (RPA) complex has been shown to be an essential player in transcription, replication, and repair [31–34]. Because of the rapid accumulation of RPA at DNA single strand breaks (SSBs) and resected DSBs, an increased number of cellular RPA foci indicates an accumulation of SSD [35]. Interestingly, a substantial rise of RPA-positive cells, as well as partial co-localization of RPA and γH2AX foci, were found 24 hours after roscovitine treatment of HPV+ UMSCC47 cells (Figure 4A and 4B). Conversely, roscovitine neither induced the formation of RPA foci, nor the colocalization of RPA and γH2AX in HPV- SCC61 cells, again suggesting that roscovitine did not induce DNA damage in HPV- cancer cells.

### Roscovitine induces DNA damage selectively in HPV+ head and neck cancer cells

Given the upregulation of γH2AX (Figures 1B and 1C, 3 and 4) and formation of RPA foci (Figure 4) after roscovitine treatment in HPV+, but not in HPV-, head and neck cancer cells, we sought to examine whether roscovitine did truly selectively induce DNA damage in HPV+ cells.

**Figure 5:**
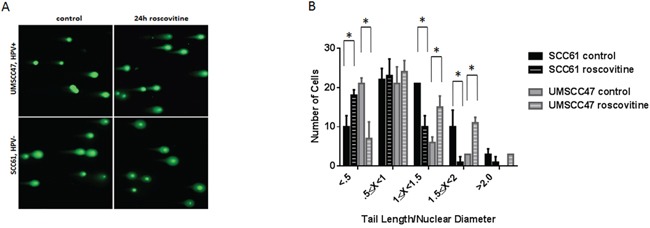
Roscovitine induces DNA damage exclusively in HPV+ head and neck cancer cells **A.** Representative images of Comet assay from HPV- SCC61 and HPV+ UMSCC47 cells untreated or treated with roscovitine for 24 hours. **B.** Quantification of Comet assay from two independent experiments.

The presence of DNA damage was determined in SCC61 and UMSCC47 cell lines using a Comet assay (Figure 5). Upon roscovitine treatment, HPV-negative SCC61 cells had a significant reduction in the average tail length/nuclear diameter ratio, corroborating with decreased H2AX phosphorylation (Figures 1B and 1D, 3 and 4), and signifying that roscovitine actually reduced the amount of damaged DNA present in SCC61 cells (Figure 5). In contrast, UMSCC47 cells showed an extensive increase in the average tail length/nuclear diameter ratio and a substantial right shift towards a higher ratio in the tail length/nuclear diameter distribution histogram, validating induction of H2AX phosphorylation (Figures 1B and 1C, and 3), and demonstrating that roscovitine treatment induced DNA damage in HPV+ UMSCC47 cells.

### Roscovitine treatment results in HPV+ cell death

**Figure 6:**
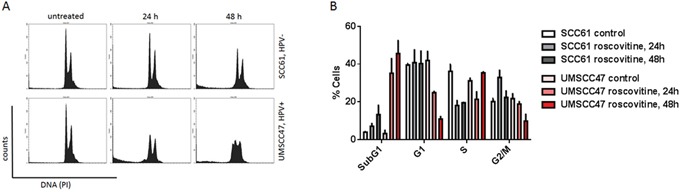
Roscovitine induces massive HPV+ cell death **A.** Cells were untreated or treated with roscovitine, collected and fixed at indicated time points, stained with propidium iodide (PI) and analyzed by flow cytometry. **B.** Percentage of cells in each phase of the cell cycle was quantified in two independent experiments.

Since roscovitine has been shown to arrest cells in the G1 and G2/M phases of the cell cycle, we then investigated if the HPV status of cancer cells would confer a different cell cycle distribution after roscovitine treatment. Fluorescence activated cell sorting (FACS) was performed on SCC61 and UMSCC47 cells treated with 20μM roscovitine for 24 and 48 hours (Figure 6). HPV- SCC61 cells experienced a time-dependent increase in the G2/M cells, decrease in the S population and a moderate increase in the sub-G1 population upon roscovitine treatment, indicating that about 16% of SCC61 cells had died 48 hours after roscovitine. In contrast, HPV+ UMSCC47 cells showed a significant decrease in the G1 population 24 and 48 hours after roscovitine application and a lesser decrease in the G2 population 48 hours after the treatment. Importantly, HPV+ cells experienced a major escalation of the sub-G1 population with about 36% and 45% of dead cells 24 and 48 hours after roscovitine treatment, respectively. Thus, roscovitine induces pronounced cell death in HPV+ cells, while transiently arresting and moderately killing HPV- head and neck cancer cells.

### Roscovitine inhibits the growth of HPV+ head and neck cancer cells *in vivo*

**Figure 7:**
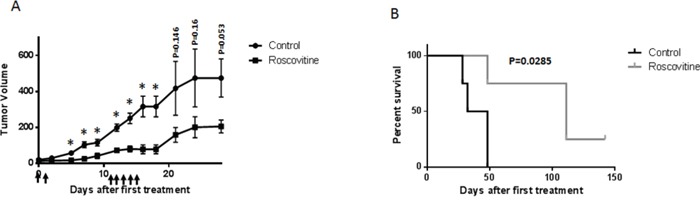
Roscovitine suppresses HPV+ tumor growth *in vivo* **A.** HPV+ UMSCC47 head and neck cancer cells were inoculated into NUDE mice. When tumors became palpable, mice were treated with 16.5 mg/kg of roscovitine or vehicle (4 mice in each group) at days indicated with arrows; tumor volume is presented. **B.** Mice were sacrificed, when tumors reached volume of 500 mm^3^, survival of mice in the control and roscovitine-treated groups is presented.

To test the potential of roscovitine as a selective agent against HPV+ head and neck cancers, a NUDE mouse-based xenograft assay was utilized. Mice were injected with HPV-positive UMSCC47 cells, and after tumors reached a measurable size, the mice were given 16.5 mg/kg doses of intraperitoneal roscovitine or vehicle injections. Tumor sizes were measured two times per week and mice were sacrificed when tumor volumes reached or exceeded 0.5 cm^3^. Roscovitine significantly reduced the rate of tumor growth (Figure 7A) and increased survival (Figure 7B) of treated mice. Strikingly, roscovitine treatment led to complete tumor disappearance in one mouse (25%); moreover, no tumor regrowth in this mouse was found 5 months after completion of the treatment (Figure 7B). Mouse weights did not differ significantly between mice treated with roscovitine and control mice, and behavioral differences between the two groups were also negligible. These results suggest that roscovitine can be used effectively as a selective tumor growth inhibitor in HPV+ head and neck cancer.

### HPV status does not determine the sensitivity of head and neck cancer cells to flavopiridol or CDK1/2 inhibitor

**Figure 8:**
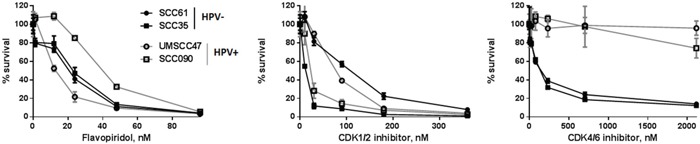
Survival after increasing doses of flavopiridol, selective CDK1/2 inhibitor III and specific CDK 4/6 inhibitor was determined in HPV-negative SCC35 and SCC61 (labeled in black) and HPV+ UMSCC47 and SCC090 (labeled in grey) head and neck cancer cell lines; standard deviations are calculated from three independent experiments

Roscovitine is selective CDK inhibitor, however, it has been shown to affect the extracellular regulated kinases, erk1 and erk2, as well as pyridoxal kinase (PDXK) that is responsible for the phosphorylation and activation of vitamin B6 [36]. To begin determining whether HPV+ head and neck cancer cells are sensitive to roscovitine due to specific CDK inhibition, we assessed the response of HPV-positive and HPV-negative cells to another broad CDK inhibitor, flavopiridol [37, 38], as well as to specific CDK1/2 [39] and CDK4/6 [40] inhibitors. HPV-positive cells are known to overexpress endogenous CDK 4/6 inhibitor p16^ink4A^; moreover, high p16 protein level is used as a surrogate marker for HPV in clinic [41]. Therefore, it was not surprising that HPV+ head and neck cancer cells were completely resistant to chemical CDK4/6 inhibitor (Figure 8). Interestingly, although cell lines, used in our study, showed different response to both, flavopiridol and CDK1/2 inhibitor, their sensitivity was not dependent on HPV status. Thus, HPV+ cells UMSCC47 displayed the highest sensitivity to flavopiridol, while another HPV-positive cell line, SCC090, was the most resistant to the same treatment (Figure 8). In contrast, UMSCC47 cells were relatively resistant to CDK1/2 inhibition, whereas SCC090 cells exhibited significantly increased sensitivity (Figure 8). These data strongly suggest that at least CDK1/2 inhibition is not responsible for the HPV-dependent sensitivity of cells to roscovitine.

## DISCUSSION

Cytotoxic drugs and radiation that are widely used in cancer therapy cause various types of DNA damage through different mechanisms of action [42]. However, systemic drug administration damages DNA not only in cancer, but also in normal healthy cells, leading to the development of severe side effects and limiting efficacy of the treatment. Therefore, drugs that cause DNA damage selectively in cancer cells will significantly improve outcomes and decrease treatment-associated morbidity, as well as reduce the instances of premature termination of therapy due to intolerance of side effects. Discovery of such drugs seems to be particularly important for patients with HPV-associated oropharyngeal squamous cell carcinoma due to two reasons. First, it is well established that these patients respond better to currently used radio- and chemotherapy, as compared to similarly staged HPV-negative head and neck cancer patients, indicating that HPV+ OPSCCs are in general more sensitive to DNA damage. Second, as no HPV status therapy de-escalation is currently used outside of clinical trials, patients treated with DNA damaging therapy are loaded with lifelong-associated morbidity that includes pronounced swallowing and speech dysfunction, mandibular osteoradionecrosis, accelerated dental decay, and lymphedema. In addition, about 20% of patients with HPV+ HNSCC suffer from recurrent cancer and distant metastases, for which effective therapies are absent.

In this study, we investigated the potential of roscovitine as a novel therapeutic agent against HPV+ HNSCC.

Roscovitine is a CDK inhibitor and antineoplastic agent that has been shown to exhibit cytotoxic effects towards multiple human cancer cells lines including colon, uterine, breast, Ewing's Sarcoma, and HPV+ cervical HeLa cells, among others [15, 18, 23, 43]. Interestingly, though roscovitine induces cell cycle arrest at the G1 and G2/M phases, previous studies reported that roscovitine appears to exert its antitumor effects by inducing apoptosis in cancer cells [12, 23, 44–47]. Roscovitine has also been associated with uncoupling replication proteins and inhibiting non-homologous end-joining DNA damage repair machinery, suggesting that the cytotoxic properties of roscovitine may be associated with the induction and/or accumulation of DNA damage [24, 25]. Though roscovitine is currently in clinical trials for a wide variety of cancers, it has never previously been suggested as an agent that selectively targets HPV+ HNSCC [11].

Here, we first determined whether the HPV status of HNSCC would confer a heightened sensitivity to roscovitine, and subsequently investigated the preliminary mechanism behind HPV status-dependent sensitivity. A NUDE mouse-based xenograft assay was also employed to test, if roscovitine had effects on tumor growth rate *in vivo.*

A clonogenic survival assay (Figure 1A) demonstrated that three HPV+ HNSCC cell lines (UMSCC47, SCC090 and SCC104) displayed a significantly increased sensitivity to roscovitine, as compared to four HPV- head and neck cell lines (SCC61, SCC35, FaDu, UNC-7). We used flow cytometry to investigate whether elevated sensitivity of HPV+ cells was due to roscovitine-induced cytotoxicity, and demonstrated that roscovitine triggered a much greater degree of cell death in HPV+ HNSCC cells, when compared to HPV- HNSCC cells (Figure 6). These results suggested that roscovitine toxicity was dependent on HPV status, and strengthened the potential of roscovitine as a selective agent against HPV+ HNSCC. Importantly, roscovitine was able to exert its selective cytotoxic effects on HPV+ HNSCC cell lines and in a xenografted mouse model (Figure 7) at relatively low concentrations, supporting its therapeutic potential in this subset of cancers, as doses could be kept low enough to minimize off-target side effects in the patient.

**Figure 9:**
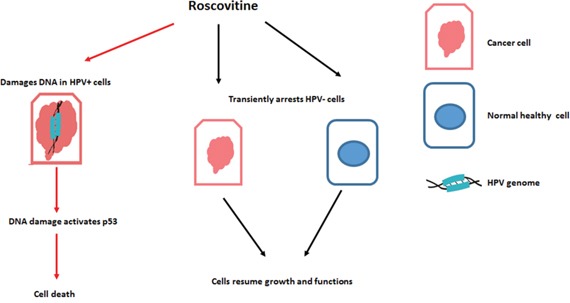
Proposed model of roscovitine selective toxicity in HPV-associated HNSCC

Roscovitine was found to upregulate the phosphorylation of H2AX in HPV+, but not in HPV- cells (Figures 1B, 1C, 1D, 3 and 4). This result corroborated previous studies that suggested that roscovitine upregulated ϒH2AX in HPV+ cancer cells [48]. Since phosphorylated H2AX is a marker of DNA damage, our findings suggested that roscovitine induces DNA damage in HPV+, but not HPV- cancer cells, which was undeniably verified utilizing the Comet assay (Figure 5), providing one possible mechanistic explanation for HPV+ HNSCC sensitivity. Interestingly, depletion of p53 with p53shRNA resulted in significant improvement of HPV-positive cells survival after the treatment with roscovitine (Figure 2A). In addition, roscovitine upregulated p53 in both HPV+ and HPV- cells (Figure 1B). Moreover, the elevated level of p53 after roscovitine treatment was transcriptionally active in HPV+ cells (Figure 2B and 2C). Cancer cells are usually very sensitive to reactivation of wild-type p53 and respond to ectopic p53 by apoptosis or growth arrest. Importantly, restoration of p53 function in established tumors results in tumor regression [49–52]. Restoring p53 expression has been suggested as a good strategy to combat HPV+ cancer. Indeed, several studies have shown that p53 stabilization in HPV+ cervical carcinoma by silencing E6 or E6AP activates the tumor suppressor function of p53 and kills cancer cells. The combination of leptomycin B and actinomycin D reduced expression of *E6* mRNA and induced apoptosis via p53 upregulation [53]. A chemical library screen identified two small molecules that suppress the growth of cervical carcinoma cells by inhibiting E6 [54]. In addition, a synthetic peptide that binds E6 and inhibits its activity has been identified [55]. The small molecule RITA [56] protected p53 from degradation and killed cervical cancer cells [57]. We found that roscovitine-induced p53 upregulation was not due to inhibition of HPV E6 (Figure 2D). We therefore suggested a model in which roscovitine selectively induces DNA damage in HPV-positive head and neck cancer cells only, which in turn, stabilizes and activates p53, finally inducing substantial HPV+ cell death (Figure 9). Our model may not completely cover all the effects of roscovitine on p53, however, since we observed induction of p53 after the treatment in the absence of DNA damage in HPV-negative cells SCC61 carrying mutant p53 (Figure 1B). However, HPV- UNC-7 cells that harbor wild type p53 were resistant to roscovitine treatment, as compared to HPV+ cells (Figure 1A), suggesting that in the absence of DNA damage, elevated p53 is either transient, or not transcriptionally active, and therefore does not stimulate HPV-negative cell death machinery. In addition, our model most likely illustrates one of several pathways that leads to selective toxicity of roscovitine in HPV+ head and neck cancer cells. We recently found that knockdown of an important player in DNA damage response, SMG-1, in cancer cells led to their increased sensitivity to roscovitine [58]; furthermore, expression of SMG-1 was diminished in HPV-positive HNSCCs due to SMG-1 promoter hypermethylation [59] that may contribute to the sensitivity of HPV+ head and neck cancer cells to roscovitine.

The exact mechanism and type of DNA damage induction by roscovitine in HPV+ cells remains unclear. It is apparent that the phosphorylation of H2AX proceeds via an ATM-independent pathway (Figure 1B), corroborating with our finding that roscovitine did not induce DNA DSBs in HPV+ cells, as indicated by the lack of p53BP1 foci formation (Figure 3). Instead, we found a significant increase in the number of RPA-positive HPV+ cells after roscovitine treatment (Figure 4), suggesting an elevated amount of single stranded DNA. Moreover, the partial overlap of RPA and ϒH2AX foci suggest the persistence of single stranded cellular DNA after roscovitine treatment. The moderate decrease in the number of cells in S phase of the cell cycle, accompanied by reduction of G1 and massive induction of cell death 24 hours after roscovitine treatment (Figure 6) suggested that roscovitine causes stalling of replication forks associated with the formation of unresolved SSD regions marked with phosphorylated H2AX. However, the exact mechanism deserves further detailed investigation. The strong HPV dependent activity of roscovitine cannot be attributed to the inhibition of CDK1/2, since the sensitivity of head and neck cancer cells to selective CDK1/2 inhibitor was not dependent on HPV status (Figure 8). Three HPV+ head and neck cancer cell lines showed similar sensitivity to roscovitine with IC50 concentrations between 2 and 3.5 μM (Figure 1A). The remarkable response pattern to broad CDK inhibitor flavopiridol, with one HPV+ cell line being the most resistant (IC50~45 nM) and another one demonstrating the significantly increased sensitivity (IC50~10 nM), together with comparable response to flavopiridol in two HPV- cell lines (IC50~22 nM) (Figure 8), suggest that selective roscovitine toxicity toward HPV-positive head and neck cancer cells may not be due to inhibition of CDKs, but most likely represent roscovitine-specific effect.

In conclusion, our study revealed selective HPV-dependent toxicity of roscovitine in head and neck cancer cells and proposed its underlined molecular mechanism. The profound HPV-positive head and neck tumor growth delaying effects of roscovitine *in vivo* further emphasize the potential of roscovitine as an anti-HPV+ HNSCC agent.

## MATERIALS AND METHODS

### Cell lines, constructs and chemicals

We used four HPV- (SCC61, SCC35, UNC7 and FaDu) and three HPV+ (SCC090, SCC104 and UMSCC47) HNSCC cell lines. All HPV- cells were cultures in (DMEM)/F12 medium supplemented with 0.4 μg/mL hydrocortizone, and all HPV+ cell lines were grown in DMEM with nonessential amino acids. All media was supplemented with 10% FBS (Invitrogen), 50 μg/mL penicillin, and 50 μg/mL streptomycin (Invitrogen). All cell lines have been tested negative for mycoplasma and microsatellites authenticated.

P-super and p-super p53 shRNA expressing vectors were a gift from Galina Selivanova.

Cells were transfected using Lipofectamine 2000 (Invitrogen) according to manufacture recommendations.

Roscovitine and specific CDK 4/6 inhibitor (PD 0332991) were obtained from Sigma. Flavopiridol and selective CDK1/2 inhibitor III were from Santa Cruz.

### Immunoblotting

Cells were collected by trypsinization and lysed in radioimmunoprecipitation assay (RIPA) lysis buffer (Sigma) with the addition of protease inhibitors (Roche) and phosphatase inhibitors (Sigma) for 30 minutes on ice. Insoluble material was removed by centrifugation at 14,000 rpm for 15 minutes at 4°C. Proteins were separated in 4% to 20% Tris-glycine polyacrylamide gels (Mini-PROTEAN; Bio-Rad) and electrophoretically transferred onto polyvinylidene fluoride membranes. Membranes were blocked with 3% BSA in PBS and incubated with antibodies against ϒH2AX and pATM (Abcam), p53 and p21 (Santa Cruz), pp53 Ser15 (Cell Signaling) and tubulin (Sigma). After incubation with primary antibodies, membranes were washed, incubated with secondary DyLight anti-mouse and anti-rabbit antibodies (Thermo Scientific), and signals was visualized using a Bio-Rad imager.

### Survival assay

All cells lines, except of SCC090 and UMSCC104, were seeded in 12-well plates at a density of 1000 cells/well in duplicates and treated with increasing doses of CDK inhibitors the following day. SCC090 and UMSCC104 were plated at a density 10,000 cells/well. After 7 days, we used Cell Titer Glo reagent (Promega) to determine the number of alive cells. The data presented in Figure 1A was obtained from 4 independent experiments.

### Immunofluorescence

Cells were grown in chamber slides, treated, fixed, immunostained, and analyzed as previously described [60]. Cells with more than 10 foci were determined as positive. The primary antibodies used were mouse anti-γH2AX (Abcam) at a dilution of 1:2,000, rabbit anti-53BP1 (Cell Signaling) at a dilution of 1:500, and rabbit anti-RPA70 (Cell Signaling) at a dilution of 1:500. Secondary anti-mouse Alexa 555 and anti-rabbit Alexa 488 were from Invitrogen and were used at a dilution 1:1000.

### Comet assay

Cells were grown in 6 well plates, treated with roscovitine, and processed for DNA damage detection using Comet Assay® Reagent Kit (Trevigen) according to their protocol. For quantification, nuclear diameter and tail length were measured in at least 50 cells using the ImageJ program.

### Fluorescent activated cell sorter (FACS)

Cells were collected by trypsin and fixed in ice-cold 70% ethanol over night at −20°C. Ethanol was removed by centrifugation and the cells were rehydrated in PBS and pelleted. The pellets were resuspended in 25 μg/ml propidium iodide (PI) (Sigma) in PBS containing 100 μg/ml RNase A (Invitrogen) and stained for 30 min at room temperature. The DNA content was analyzed by FACSCalibur flow cytometer (BD Biosciences). Samples were gated on the single cell population, and 10,000 cells were collected for each sample.

### RNA extraction and quantitative RT-PCR

Total RNA was extracted by Qiagen RNA extraction kit and cDNA was synthesized using iScript cDNA Synthesis Kit (Bio-Rad) according to the manufacturer's instructions. Quantitative real-time reverse transcription (qRT-PCR) was done using iQ SYBR Green Supermix (Bio-Rad) and primer pairs: CDKN1A from Origene; Forward 5′AAGCAACAGTTACTGCGACGTGAG3′ and Reverse 5′ CGGTCCACCGACCCTTATATT3′ for HPV16 E6; Forward 5′ ACCGGACAGAGCCCATTACA3′ and Reverse 5′ GCCCATTAACAGGTCTTCCAA3′; Forward 5′ AGGGCTGCTTTTAACTCTGGT3′ and Reverse 5′ CCCCACTTGATTTTGGAGGGA3′ for human GPDH; on the iCycler iQ Real-Time PCR Detection System (Bio-Rad). Each qRT-PCR reaction was done in at least duplicate, and the ΔΔCt method was used to analyze the data.

### *In vivo* experiments

The *in vivo* study was approved by the local animal experimental ethical committee. Exponentially growing UMSCC47 cells were injected subcutaneously into the sacral area of female NUDE mice. Each mouse was inoculated with 2 × 10^5^ cells in 50% matrigel and 50% PBS at a volume of 100 μL. Body weight, tumor growth, and general behavior were monitored. Tumor volumes were measured every 3 days. Mice were sacrificed when the tumor exceeded a size of 0.5cm^3^.

### Statistical analysis

The Kaplan–Meier method was used to generate survival curves, and log-rank test analysis was used to compare roscovitine-treated and untreated mouse groups. Other statistical analyses were done using Fisher exact and χ2 for trend tests.
